# The Future Tuberculosis Institution Resident

**Published:** 1913-10-18

**Authors:** 


### THE FUTURE TUBERCULOSIS INSTITUTION RESIDENT.

Since pulmonary phthisis began to be treated in sanatoriums, it is noticeable (although the fact has eluded comment, and may provoke hasty criticism) how many of their residents have been recognised as clinical authorities. We will leave out of count Brehmer, because he, like our own Bodington, owed his fame to his position as a pioneer. But there is Trudeau, quoted in Cornet's standard work; Turban, whose clinical classification of the disease is in use all over the world; Carl Spengler, to whom Koch entrusted the therapeutic trial of tuberculin; Moeller, given the title of Professor, and selected as contributor to the great compilation on German clinical work at the outset of the twentieth century; Saugmann, secretary of an international medical society, and contributor to the sole existent system of the therapeutics of consumption; and Bandelier, collaborator with another sanatorium resident in two works on tuberculosis which have attained several editions and translations. All these names, as will be noticed, are foreign ones; indeed, it is abroad that the tendency mentioned is shown most. However, signs exist that things are beginning to go the same way at home. The composition of the recent Departmental Committee upon Tuberculosis affords a good instance; so does the very practical recognition of Dr. Paterson's "Arbeitskur," and his appointment to organise the anti-tuberculosis campaign in Wales. And with the increase in tuberculosis institutions which has now begun, the rate of progress in this direction is accelerating.

### Education and Training.

But let us examine not only the direction of the trend, but also its point of departure. In the past the tuberculosis institution resident was either a house physician at a consumption hospital, or a house surgeon at a hip disease one. He was quite young; he stopped six months or, at most, a year; he had no notion of finding his life work in the treatment of tuberculosis, the vacancies on the senior staff being filled up by others—physicians or surgeons of general hospitals. When sanatorium treatment came in, it had to be adapted somewhat to this constitution of staff. The discipline and minute supervision of patients necessitated, it is true, more responsible resident managership, and therefore medical superintendentships and resident medical officerships replaced the short service appointment. The gentlemen filling these newer posts did not, however, find themselves out of leading strings. They were not often present at house-committee meetings, or entrusted with the selection of patients; they had visiting "honoraries" to entertain—until the latter came to recognise rather generally that the post of honorary medical visitant to a sanatorium did not present much scope for energy. Meanwhile the tuberculosis problem was increasing rapidly in complexity: economically, to begin with, it being recognised that everything must come down to pounds, shillings, and pence if the best were to be done for the consumptives of a neighbourhood, and crippled children to get some sort of an education; medically too, for laboratory work was called for, and laryngology, and treatments with a time-demanding technique were coming in; administratively—larger institutions requiring thought and the sempiternity of the master's eye more than small ones; bibliographically even—for tuberculosis had now developed an immense and polyglot literature which well repaid study. Anyone who has visited the scene of Dr. Gauvain's daily work in Hampshire will realise all these points, and more also.

### Post-Graduate Specialisation.

And so it seems to come to this, that as homogeneity goes on passing, here as elsewhere, into heterogeneity, the different fields of study become capable of more intensive cultivation, and whole-time workers are called for. Whole-time tuberculosis workers, such as those we have mentioned, or of the type of Dr. Morland, of Arosa, are what the current tuberculosis movement in this country really needs more than anything else. Besides the immediate effect, they would train assistants to become real phthisiologists like themselves. For just as one cannot learn to swim on dry land, so general academic accomplishment, however routinely excelling, is no means to special proficiency. What counts towards that is, as every doctor knows, post-graduate experience. When it comes to the percentage of practical successes in diagnosis or treatment, the man who has seen his thousand cases will surpass the M.D. who has put in six months at tuberculosis work with a view to becoming a tuberculosis officer; will surpass him very noticeably. The best way, physiology now decrees, to make a soldier a good marcher is to give him marching to do—not gymnasium physical exercises, even of the most fashionable kind. Similarly, close and prolonged and continuous familiarity with a special clinical material is the first requisite to becoming a real expert. This movement, this trend of things, may seem hard upon the general physician, but yet, after all, what final validity have the existing divisions of convenience of medical work, save in so far as they are heuristic and beneficent? And if the general physician has suffered other encroachments in the past, as from the surgeon and the bacteriologist, it was only because his field, in the light of the latter-day expansion of medical as of other knowledge, was seen to be unmanageably big. He has his remedy; already there is cardiology, for instance. At any rate, with him too, progress must have its way; intensive must go on taking from extensive culture.

---

**H.R.H. The Duchess of Albany has graciously consented to open the extension of the Florence Nightingale Hospital for Gentlewomen, 19 Lisson Grove, N.W., on Friday, November 14.**

